# Interplay of replication stress response and immune microenvironment in high-grade serous ovarian cancer

**DOI:** 10.3389/fcell.2025.1638964

**Published:** 2025-09-05

**Authors:** Laura Venegas, Stephanie Lheureux

**Affiliations:** Division of Medical Oncology and Hematology, Princess Margaret Cancer Centre, University Health Network, Toronto, ON, Canada

**Keywords:** TME (tumor microenvironment), replication stress, HGSOC, Wee1, Chk1, ATR, immunotherapy, PDL1 inhibitors

## Abstract

High-grade serous ovarian cancer (HGSOC) is the most lethal gynecological malignancy. Therapeutic options remain limited for patients lacking predictive biomarkers, particularly those with BRCA wild-type tumors or those who have acquired resistance to both PARP inhibitors and platinum-based chemotherapy. Replication stress, TP53 mutations, and genomic instability characterize HGSOC. The cellular response to replication stress is primarily mediated by checkpoint kinases; however, this mechanism is frequently impaired in tumor cells. Consequently, cancer cells become increasingly dependent on the replication stress response (RSR) pathway for survival, and susceptible to therapies targeting the ATR-CHK1-WEE1 axis—a key regulator of genomic integrity. Inhibition of these checkpoint kinases can disrupt cell cycle control, inducing mitotic catastrophe and subsequent cancer cell death. Another defining feature of HGSOC is its immunosuppressive tumor microenvironment (TME), which has limited the efficacy of immune checkpoint inhibitors. Emerging evidence suggests that inhibition of the RSR pathway may not only exploit intrinsic tumor vulnerabilities but also modulate the TME to enhance anti-tumor immune responses. This provides rationale for combination approaches integrating RSR pathway inhibitors with innovative immune checkpoint blockade (ICB). This review examines the mechanistic rationale and therapeutic potential of such combinations, drawing on both preclinical and clinical data.

## 1 Introduction

DNA damage induces replicative stress, a critical cellular alteration that can arise from exogenous agents—such as cytotoxic chemotherapies (e.g., gemcitabine, 5-fluorouracil, cisplatin)—or endogenous factors, including misincorporation of ribonucleotides or mutations in tumor suppressor genes (Zeman and Cimprich, 2014). The cellular response to replicative stress is a regulated mechanism that ensures accurate DNA replication and genome integrity (Saxena and Zou, 2022). In tumor cells, this response becomes essential for survival; when compromised, tumor cell proliferation is impaired (Schoonen et al., 2019).

Ovarian cancer is the most lethal gynecologic malignancy, responsible for approximately 207,000 deaths worldwide each year (Huang et al., 2022). HGSOC is the most common histology subtype and is characterized by genomic instability, universal TP53 mutations, and profound copy number changes (Hillmann et al., 2025; Cancer Genome Atlas Research Network, 2011). The loss of the tumor suppressor gene p53 promotes a sequential pattern of genomic instability as tumors evolve. This progression begins with the accumulation of deletions, particularly in p53, and copy number alterations, followed by genome doubling and subclonal expansion, leading to intratumoral heterogeneity that contributes to poor prognosis and treatment resistance (Baslan et al., 2022). In a preclinical model using cell lines derived from non-ciliated fallopian tube epithelial cells, TP53 mutation appears to act as an initiating event, while subsequent BRCA1 loss further increases chromosomal instability (CIN) (Bronder et al., 2021). These molecular alterations coincide with progressive changes in the TME, transitioning from immune surveillance in early serous tubal intraepithelial carcinomas (STICs) to immune suppression in advanced STICs and cancer (Kader et al., 2024). The loss of p53 also upregulates repetitive elements, triggering an antiviral immune response known as viral mimicry; however, in premalignant lesions, this response becomes progressively suppressed, contributing to the development of immune tolerance (Ishak et al., 2025). Another contributor to the progressive cascade of events is the amplification of Cyclin E1 (CCNE1), which accelerates the transition into synthesis phase (S phase), increases cellular proliferation, and exacerbates replication stress (Aziz et al., 2019).

A major therapeutic discovery in HGSOC has been the introduction of Poly(ADP-ribose) polymerase 1/2 inhibitors (PARPi), which have shown clinical benefit predominantly in patients with defects in DNA damage repair pathways based on the concept of synthetic lethality (Farmer et al., 2005; Konstantinopoulos et al., 2015). More recently, the inhibition of cell cycle–regulating kinases has emerged as an interesting treatment strategy. These agents are currently under investigation and have demonstrated encouraging activity, particularly in a selective group of patients, including CCNE1 amplified tumors (Xu et al., 2024). However, patients with no identified biomarker, such as BRCA mutation, homologous recombination deficiency (HRD) phenotype, or CCNE1 amplification, face a biological challenge with limited therapeutic options, representing a significant unmet need (Wang YW. et al., 2025). This underscores the importance of identifying novel target therapies or rational combination strategies for this population beyond genomic alterations. Efforts to improve clinical outcomes using anti-PD(L)1 therapies—either as monotherapies or in combination with PARPi or chemotherapy—have mainly failed, demonstrating limited efficacy across multiple clinical trials. (Ghisoni et al., 2024). Emerging evidence suggests that modulation of the TME and inhibition of kinases involved in the replicative stress process could enhance therapeutic efficacy (Hardaker et al., 2024a). However, a deeper mechanistic understanding of these interactions is still needed. This review explores the interaction between replicative stress and the TME and summarizes current preclinical and clinical evidence supporting the combination of cell cycle checkpoint inhibitors with anti-PD(L)1 therapy in HGSOC. Our literature review is narrative in nature rather than a systematic review, we included preclinical original research, and clinical trials relevant for the topic, non-english publications or non-peer-reviewed materials were excluded.

## 2 Intercommunication between replication stress and the immune microenvironment

**FIGURE 1:**
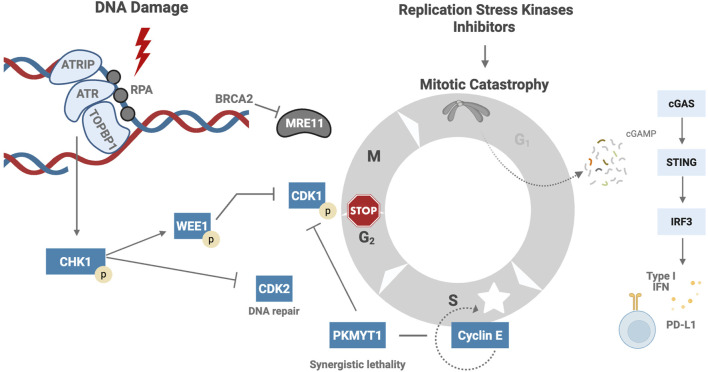
Cellular Response to Replication Stress and Immune Activation. RPA binds to ssDNA at the stalled fork and recruits ATR kinase through its partner ATRIP. ATR activation is facilitated by TopBP1 and leads to Chk1 phosphorylation (Saldivar et al., 2017). Activated Chk1 induces cell cycle arrest at the S-G2 phase by downregulating CDK2 and delaying S-phase progression. Chk1 also activates WEE1, which inhibits CDK1, enforcing G2-M arrest and stabilizing the replication fork by preventing nuclease-mediated degradation (Saxena and Zou, 2022). BRCA2 plays a critical role in protecting stalled forks from degradation by the MRE11 nuclease (Oh and Symington, 2018). In the absence of efficient checkpoint signaling, cells may enter mitosis prematurely, resulting in micronuclei formation. DNA fragments within the cytoplasm activate the cGAS-STING pathway, leading to transcription of type I interferon genes and PDL1 overexpression (Tong et al., 2024; Sato et al., 2017). ssDNA – Single-stranded DNA, ATR – Ataxia telangiectasia and Rad3-related protein, ATRIP – ATR Interacting Protein, TopBP1 – Topoisomerase II Binding Protein 1, Chk1 – Checkpoint kinase 1, CDK2 – Cyclin-dependent kinase 2, S-G2 phase – Synthesis to Gap 2 phase of the cell cycle, WEE1 – WEE1 G2 checkpoint kinase, CDK1 – Cyclin-dependent kinase 1, BRCA2 – Breast Cancer gene 2, MRE11 – Meiotic recombination 11 homolog, cGAS – Cyclic GMP-AMP synthase, STING – Stimulator of interferon genes, PDL1 (or PD-L1) – Programmed death-ligand 1. Created in BioRender. venegas, l. (2025) https://.BioRender.com/58mfsso.

DNA replication, under normal conditions, occurs in an organized and coordinated manner, ensuring that DNA is replicated only once and is equally distributed to the daughter cells (Sørensen and Syljuåsen, 2012). However, various factors can disrupt this delicate process, leading to replication stress. Some causes of replication stress include the release of reactive oxygen species (ROS), incorrect incorporation of ribonucleotides, alterations in DNA structure, or collisions between the transcription and replication machinery (Zeman and Cimprich, 2014). In response to this stress, a cascade of proteins is activated (Figure 1), starting with the replication protein A (RPA), the initial sensor that binds to single-stranded DNA (ssDNA) at the stalled replication fork and recruits ATR kinase. Subsequently, ATR kinase collaborates with Interacting Protein (ATRIP), activated by Topoisomerase II Binding Protein 1 (TopBP1). Once activated, ATR phosphorylates Checkpoint kinase 1 (Chk1), which induces cell cycle arrest at the S-G2 phase, providing time for DNA repair mechanisms to act, including homologous recombination (HRR) and non-homologous end joining (NHEJ) pathways. In addition, Chk1 regulates the G2-M transition by reducing cyclin-dependent kinase 2 (CDK2), slowing replication in the S phase. Chk1 also phosphorylates and activates WEE1, which negatively regulates cyclin-dependent kinase 1 (CDK1), also known as CDC2, resulting in cell cycle arrest, which is essential for entry into mitosis. WEE1 also stabilizes the replication fork by inhibiting nucleases and preventing DNA degradation (Domínguez-Kelly et al., 2011). Other participants in the DNA damage response (DDR) include BRCA2; Its function is to protect the replication fork from degradation by MRE11 nuclease (Oh and Symington, 2018).

In cancer, replication stress is particularly prevalent due to the loss of function of tumor suppressor genes like TP53, RB1, and NF1 (Khamidullina et al., 2024). HGSOC exhibits genomic complexity, and analysis of copy number alterations has identified seven signatures. Signature 1 is associated with breakage–fusion–bridge (BFB) cycles and active RAS signaling; Signature 4 correlates with whole-genome doubling and amplification of CCNE1 and MYC; and Signature 6 is characterized by aberrant G1/S cell cycle checkpoint control (Macintyre et al., 2018). In response to the replication stress induced by DNA damage, the ATR-CHK1-WEE1 axis is crucial for the survival and proliferation of cancer cells (Gaillard et al., 2015). However, in tumor cells, cell cycle regulation is abnormal, and the cell may proceed to mitosis with unrepaired DNA damage, ultimately leading to mitotic catastrophe, cell death, and micronuclei formation (Dobbelstein et al., 2015; Zhang et al., 2022; Kwon et al., 2020). These DNA fragments are released into the cytoplasm, where the cyclic GMP-AMP synthase (cGAS) sensor recognizes the self-derived DNA in the cytosol, leading to the production of cyclic guanosine monophosphate–adenosine monophosphate (cGAMP), a second messenger, which activates stimulator of interferon genes (STING) and triggers the transcription of type 1 interferon-related genes. This pro-inflammatory signal promotes an anti-tumor immune response and upregulates programmed cell death ligand 1 (PD-L1) expression (Tong et al., 2024; Sato et al., 2017).

## 3 Targeting replication stress in ovarian cancer

Numerous clinical trials have investigated the potential of inhibiting kinases involved in replication stress, such as ATR, Chk1, and WEE1, in HGSOC. However, efficacy was modest as a single agent in not selected patients, with objective response rates (ORR) in platinum-resistant ovarian cancer (PROC), ranging from 5% to 15% and 20%–25% in selected patients with sensitive alterations, such as ataxia telangiectasia (ATM) mutations and CCNE1 amplification. Response rates tend to improve when combined with chemotherapy or PARPi; however, hematologic toxicity remains a major limitation (Supplementary Table S1) (Yap et al., 2024; Tan et al., 2022; Yap et al., 2023; Shah et al., 2021; Simpkins et al., 2024; Mahdi et al., 2021; Konstantinopoulos et al., 2024; Konstantinopoulos et al., 2022; Giudice et al., 2024a; Kristeleit et al., 2023; Jones et al., 2023; Miller et al., 2022; Fu et al., 2023; Westin et al., 2021; Lheureux et al., 2021; Moore et al., 2022; Leijen et al., 2016; Au-Yeung et al., 2022; Oza et al., 2020; Liu et al., 2023; Gelderblom et al., 2023; Schram et al., 2025).

## 4 PARP inhibition and immune regulation

**FIGURE 2:**
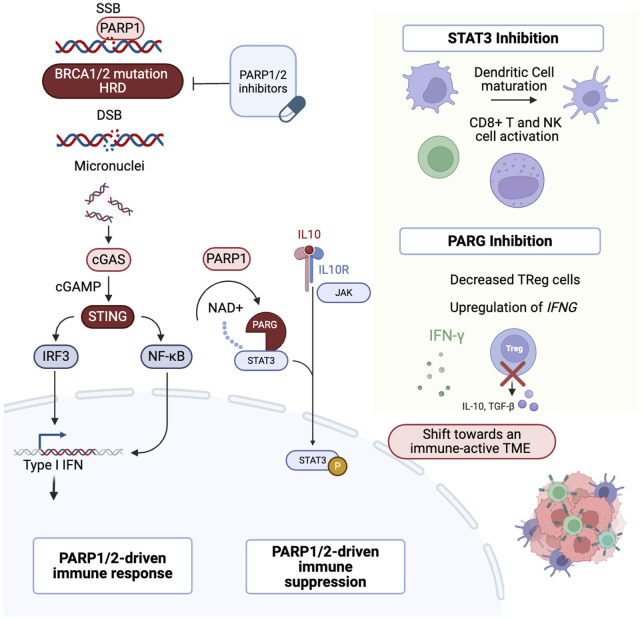
Multifaceted role of PARP1. After DNA damage, PARP1 binds to SSBs. In cells with HRD, PARP1/2 inhibition leads to DSBs. DNA fragments are then released into the cytoplasm and recognized by the cGAS sensor, activating the cGAS–STING pathway. This triggers IRF3 translocation to the nucleus and activates the IFN response. In addition, PARP1 modulates STAT3 through PARylation, promoting an immunosuppressive TME. Inhibition of STAT3 or PARG can shift the TME towards an immune-active state (Zhu et al., 2021; Dunphy et al., 2018; Shen et al.; Yue et al., 2012; Z et al., 2025; Martincuks et al., 2021a; Yu et al., 2007; Ding et al., 2019; Houl et al., 2019; Martincuks et al., 2024). PARP1/2: Poly (ADP-ribose) polymerase 1 and 2, SSBs: Single-Strand Breaks, HRD: Homologous Recombination Deficiency, DSBs: Double-Strand Breaks, cGAS: cyclic GMP-AMP synthase, STING: Stimulator of Interferon Genes, IRF3: Interferon Regulatory Factor 3, IFN: Interferon, STAT3: Signal Transducer and Activator of Transcription 3, TME: Tumor Microenvironment, PARG: Poly (ADP-ribose) glycohydrolase. Created in BioRender. venegas, l. (2025) https://BioRender.com/p3z3594.

The interaction between PARP1/2 inhibition and the cGAS-STING pathway has driven clinical trials investigating the use of PARPi and anti-PD-(L)1 therapies in HGSOC (Ghisoni et al., 2024). PARP inhibition leads to the accumulation of cytosolic DNA, which is recognized by cGAS. This recognition activates the STING pathway in the endoplasmic reticulum. Upon activation, STING recruits TANK-binding kinase 1 (TBK1), which activates transcription factors such as interferon regulatory factor 3 (IRF3) and nuclear factor kappa B (NF-κB). These factors translocate to the nucleus and induce the expression of genes involved in modulating the immune response (Figure 2) (Zhu et al., 2021; Dunphy et al., 2018; Shen et al.).

Additional mechanisms of interaction of PARP inhibition with the TME have been studied in preclinical models. However, further clinical validation is needed. PARP1/2 inhibition activates signal transducer and activator of transcription 3 (STAT3), a key factor implicated in immune evasion and treatment resistance (Yue et al., 2012; Z et al., 2025), by inhibiting TH1-type immune responses and promoting the overexpression of IL-6, IL-10, and VEGF, which contributes to an immunosuppressive TME (Martincuks et al., 2021a). Poly (ADP-ribose) glycohydrolase (PARG) is an enzyme that counteracts PARP by reversing the PARylation process (Houl et al., 2019). PARG inhibition decreased pSTAT3 levels *in vitro* and promoted antitumor immunity *in vivo* by increasing interferon-gamma expression, activating CD8^+^ T cells, and reducing the population of regulatory T cells (Martincuks et al., 2024). Despite the preclinical rationale, this has not yet been translated into the clinic. One limitation is the lack of models that accurately replicate the dynamic interactions between the tumor and the immune microenvironment, reflecting the evolving genomic and immune landscape (Stur et al., 2025).

Clinical trial results combining anti-PD(L)1 and PARP1/2i are inconsistent, and to date, none of these combinations have been approved for clinical practice in HGSOC (Ghisoni et al., 2024). While the triple combination of durvalumab, olaparib, and cediranib did not improve progression-free survival (PFS) (Lee et al., 2025), the combination of olaparib, durvalumab, and bevacizumab demonstrated encouraging results in patients without BRCA mutations (Drew et al., 2024). Therefore, the prolonged response observed in some patients warrants further investigation to better understand the interactions between the TME and the DNA repair pathways (Fu et al., 2023).

Resistance to PARP1/2 inhibitors is frequent (Soberanis P et al., 2023), and preclinical studies have shown that these inhibitors can interact differently with replication stress (Shih et al., 2024). Replication stress kinase inhibition have been explored in clinical trials, either as monotherapy or in combination with PARP1/2i, as a strategy to overcome resistance; however, modest response rates emphasize the need for novel therapeutic combinations (Stur et al., 2025). The contribution of tumor-extrinsic factors, particularly the role of TME, to acquired therapeutic resistance represents an important area of investigation.

## 5 Modulating immune response through replication stress kinase inhibition

**FIGURE 3:**
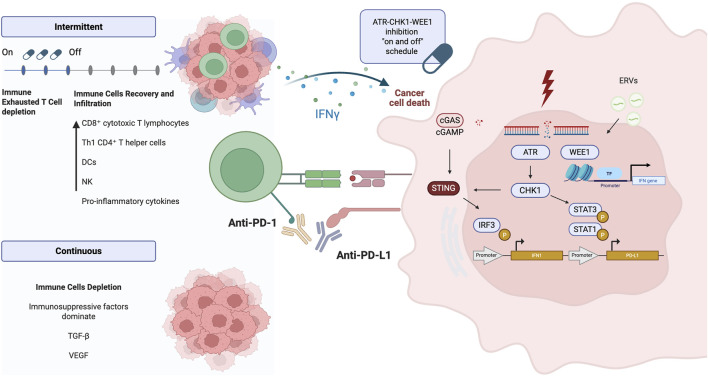
ATR-CHK1-WEE1 pathway and immune interaction. DSBs activate the ATR–Chk1 pathway, which subsequently phosphorylates STAT1 and STAT3, leading to the overexpression of PD-L1. In parallel, the cGAS–STING pathway promotes the transcription of type I interferon genes in response to cytosolic DNA. WEE1 kinase modulates interferon gene expression through recognition of ERVs and regulation of chromatin. An intermittent dosing schedule of ATR, Chk1, and WEE1 inhibitors allows immune cell recovery, enhances immune cell infiltration, and promotes activation of anti-tumor immune responses (Sato et al., 2017; Carlsen and El-Deiry, 2022; Iwai et al., 2017). DNA: Deoxyribonucleic Acid, DSBs: Double-Strand Breaks, ATR: Ataxia Telangiectasia and Rad3-related protein, Chk1: Checkpoint kinase 1, STAT1: Signal Transducer and Activator of Transcription 1, STAT3: Signal Transducer and Activator of Transcription 3, PD-L1: Programmed Death-Ligand 1, cGAS: Cyclic GMP-AMP Synthase, STING: Stimulator of Interferon Genes, WEE1: WEE1 G2 Checkpoint Kinase, ERVs: Endogenous Retroviral Elements. Created in BioRender. venegas, l. (2025) https://BioRender.com/kedtvdt.

Preclinical evidence has shown that inhibiting kinases involved in replication stress can modulate the immune response (Figure 3) (Taniguchi et al., 2024).

In an *in vivo* colorectal cancer mice model, the ATR inhibitor M6620 (VX-970), when combined with cisplatin, carboplatin, or irinotecan and the anti–PD-L1 antibody avelumab, demonstrated significant anti-tumor activity; similarly, in the MB49 urothelial tumor model, the combination of carboplatin and avelumab also exhibited therapeutic efficacy (Alimzhanov et al., 2020).

An intermittent schedule, in a colorectal cancer mice model, ceralasertib 7 days on, 7 days off, combined with the anti-PD-L1 antibody durvalumab, significantly improved survival through a CD8^+^ T-cell-dependent mechanism. The intermittent schedule led to superior tumor control compared to continuous treatment. CyTOF and scRNAseq analysis of the TME revealed that ceralasertib reshapes the TME by decreasing the exhausted CD8^+^ T-cell phenotype and reducing monocytic myeloid derived suppressor cells (M-MDSCs) and tumor-associated macrophages (TAMs). Additionally, ceralasertib increased the presence of CD11c+ MHC II + dendritic cells (DCs). While low-dose ceralasertib showed minimal or no anti-tumor effect *in vitro* or *in vivo* when used alone, its combination with PD-L1 blockade resulted in significant anti-tumor activity (Hardaker et al., 2024b).

The Chk1 inhibitor prexasertib (12 mg/kg, BID, 2/7 days) elicited a immune-mediated anti-tumor response in both *in vitro* and *in vivo* in Small Cell Lung Cancer (SCLC) models. Treatment with prexasertib induced dynamic remodeling of the TME, characterized by increased infiltration of CD3^+^ total T cells and CD8^+^ cytotoxic T cells, and reduction in exhausted T cells by day 7. When combined with anti–PD-L1 therapy (300 μg, administered once weekly on day 3), prexasertib significantly enhanced therapeutic efficacy. Mechanistically, this immune activation was associated with activation of the cGAS–STING–TBK1–IRF3 signaling axis, leading to induction of type I interferon responses, upregulation of PD-L1 expression, and CXCL10 and CCL5 cytokines (Sen et al., 2019a).

The combination of the Chk1 inhibitor SRA737, anti-PD-L1, and low-dose gemcitabine (LDG) was assessed in a SCLC model. While no significant anti-tumor activity was observed with any of the single-agent treatments, the combination led to substantial tumor regressions. Flow cytometry analysis demonstrated a significant increase in CD3^+^ and CD8^+^ T-cell infiltration compared to vehicle or single-agent treatments, and a reduction in CD4^+^ helper T-cells, regulatory T-cells, and exhausted CD8^+^ T-cells. The combination therapy increased M1 macrophage populations and DCs, while decreasing M2 macrophages and MDSCs (Sen et al., 2019b).

In ovarian cancer cell lines, the WEE1 inhibitor AZD1775 modulates the immune response by inducing expression of endogenous retroviral elements (ERVs), which produces double-stranded RNA (dsRNA), activating IFN-mediated anti-tumor signaling and upregulating PD-L1. This effect was driven by downregulation of the histone H3K9me3. *In vivo*, STING-deficient ID8 ovarian cancer mice model, AZD1775 (5 days on, 2 days off) combined with anti–PD-L1 antibody significantly enhanced anti-tumor efficacy (Guo et al., 2022).

In a phase I clinical trial involving patients with advanced solid tumors, Prexasertib, in combination with the PD-L1 inhibitor LY3300054, exploratory analysis of immune cell samples collected before and after treatment revealed significant increases in activated CD8^+^ T cells and natural killer T cells following treatment. Of the 17 patients enrolled, 10 had high-grade serous cancer. The majority of patients exhibited notable signs of T-cell activation (Do et al., 2021).

In a Phase II clinical trial of prexasertib monotherapy in BRCAwt, platinum-resistant HGSOC, exploratory analysis of immune cell subsets revealed that patients with non-clinical benefit exhibited an increase in M-MDSCs, while patients with clinical benefit showed decreased expression of immune suppressive marker TIM-3 on CD8^+^ Tregs (Giudice et al., 2024b).

These findings are primarily based on non-ovarian models across various solid tumors, where the TME differs from that of HGSOC. Clinical evidence is limited, as the interaction between CHK1 inhibition and TME modulation is derived from a single phase 1 clinical trial. These results require further validation through dedicated models in ovarian cancer.

## 6 Inhibition of replication stress kinases and anti-PD(L)1

Most clinical trials investigating PD-(L)1 inhibitors combined with ATR, WEE1, or Chk1 inhibitors have been performed in non-ovarian cancers, which have different TME (Kim et al., 2022; Besse et al., 2024; Kwon et al., 2022; Brond et al., 2021). These studies have focused on tumor types with established sensitivity to immune checkpoint blockade, such as melanoma and small cell lung cancer. Supplementary Table S2 summarizes ongoing clinical trials evaluating the combination of ATR inhibitors and immune checkpoint inhibitors across multiple tumor types, many of which are still actively enrolling participants, highlighting sustained interest in this therapeutic strategy.

To date, results have been reported from four trials (Table 1). Two of these specifically investigated the potential of ATR inhibitors to overcome resistance to ICB, and one study included patients with HGSOC (Kim et al., 2022; Besse et al., 2024; Kwon et al., 2022; Brond et al., 2021).

**TABLE 1:** Reported clinical trials investigating ATR or Chk1 inhibition and anti-PDL1 therapy in solid tumors.

Study	Population	Treatment	ORR	Median PFS (months)	Median OS (months)	Exploratory correlatives
Phase 2 metastatic melanoma^79^	N = 30 prior anti-PD-1	Ceralasertib + Durvalumab	31%	7.1	14.2	Better outcomes in immune-enriched TME & DDR alterations; trend for improved PFS in HRD tumors (HR 0.17; P = 0.064). Responders showed higher MHC-I, Treg, IFN signatures
Phase 2 HUDSON NSCLC umbrella study^80^	N = 268 NSCLC patients post anti-PD-(L)1 & platinum	Durvalumab + Ceralasertib (n = 79) vs. other regimens (n = 189)	13.9% vs. 2.6%	5.8 vs. 2.7	17.4 vs. 9.4	ATM alterations: ORR 26.1%, PFS 8.4 mo, OS 22.8 mo. CDKN2A alterations linked to shorter PFS. Biomarker data showed enhanced TCR diversity post durvalumab and ceralacertib
Phase 2 advanced gastric cancer^74^	N = 31	Ceralasertib + Durvalumab	22.60%	3	6.7	DDR gene mutations enriched in responders (p = 0.022); HRD associated with prolonged PFS (HR 0.13; p = 0.0002), especially with ATM loss/high HRD score
Phase 1 advanced solid tumors^81^	N = 17 patients (14 ovarian cancer)	Prexasertib (CHK1i) monotherapy, and LY3300054 (anti-PD-L1) combination	Partial responses in 50% CCNE1-amplified HGSOC patients	NA	NA	CCNE1 amplification in 6 patients; 3 had PRs (response durations 7, 13, 20 mo), 1 had durable SD > 12 mo. Increased activated CD8^+^ T cells (CD71^+^)

NSCLC: Non-Small Cell Lung Cancer, HGSOC: High-Grade Serous Ovarian Cancer, PD-(L)1: Programmed Death-(Ligand) 1, TME: tumor microenvironment, DDR: DNA, damage response, HRD: homologous recombination deficiency, MHC-I: Major Histocompatibility Complex Class I, Treg: Regulatory T Cell, IFN: interferon, TCR: T Cell Receptor, SD: stable disease, PR: partial response, OS: overall survival, PFS: Progression-Free Survival, ORR: objective response rate, CI: confidence interval, HR: hazard ratio, mo: months, ATM: ataxia telangiectasia mutated, CDKN2A: Cyclin Dependent Kinase Inhibitor 2A, CCNE1: Cyclin E; EOC: Epithelial ovarian cancer.

In a Phase II study of 30 patients with metastatic melanoma who had progressed on prior anti–PD-1 therapy, the combination of ceralasertib and durvalumab demonstrated an ORR of 31% (95% CI, 13.6%–46.4%), a median PFS of 7.1 months (range, 3.8–11.7) and a median overall survival (OS) of 14.2 months (95% CI, 9.3–19.1); 44.4% patients with primary resistance achieved a response. Exploratory biomarker analyses demonstrated that patients with an immune-enriched TME or alterations in the DDR pathway derived the greatest benefit. Responders exhibited a higher expression of major histocompatibility complex class I (MHC-I) and interferon-related gene signatures (Kim et al., 2022). Similarly, in the phase 2 HUDSON umbrella study in patients with NSCLC post anti-PD-L1 and platinum-doublet therapy, durvalumab–ceralasertib combination demonstrated superior efficacy compared to other regimens; responses were particularly pronounced in patients with ATM alterations, correlative biomarker analyses revealed downregulation of monocyte, CD8^+^ T cell, and exhaustion-associated gene signatures along with upregulation of TNF-α, interferon-γ, and interferon-α pathways (Besse et al., 2024). Based on these findings, LATIFY (NCT05450692) is an ongoing phase III, open-label, randomized, multicenter trial evaluating the efficacy and safety of ceralasertib plus durvalumab *versus* docetaxel in patients with locally advanced or metastatic NSCLC who have progressed after anti–PD-(L)1 therapy and a platinum-based doublet.

In a phase II study, patients with advanced gastric cancer treated with ceralasertib and durvalumab demonstrated an ORR of 22.6%, mPFS of 3.0 months, and mOS of 6.7 months; only 6.5% have received prior anti-PD1 therapy. Whole-exome sequencing of pretreatment tumor biopsies revealed enrichment of mutations in DDR pathway genes among patients who achieved partial responses, and HRD was associated with prolonged PFS. Correlative analyses showed that responders exhibited an increase in intratumoral lymphocyte infiltration and expansion of circulating tumor-reactive CD8^+^ T-cell clones. In contrast, treatment resistance was associated with enriched tumor vasculature signatures and decreased T-cell receptor (TCR) clonality (Kwon et al., 2022).

Prexasertib, a Chk1 inhibitor, and anti-PD-L1 LY3300054 were evaluated in a Phase I study, anti-PD-L1 monotherapy, or combination. The study included 14 patients with recurrent ovarian cancer. The most common histology was HGSOC. CCNE1 amplification was present in six patients, 50% achieved PR (Do et al., 2021).

## 7 Discussion

We summarize how the ATR-CHK1-WEE1 signaling axis is critical for maintaining genomic stability and how cancer cells often rely on this pathway for survival. Therefore, the development of drugs targeting these cell cycle checkpoint kinases is of interest and has shown some encouraging results in cancer treatment. While the molecular mechanisms of this pathway are well understood, its connection to the TME remains poorly characterized. Emerging evidence suggests that modulation of the immune response through inhibition of these kinases, particularly via the cGAS-STING pathway and STAT1/STAT3 transcription factors, which activate a type I interferon response and upregulate PD-L1, contributes to anti-tumor immunity (Sato et al., 2017; Taniguchi et al., 2024). However, other players, such as PARG and epigenetic regulators (Martincuks et al., 2024), may also be involved.

Chromosomal instability in HGSOC arises from cumulative alterations in cell cycle regulators, rather than from a single genetic alteration or mutation, which accumulates over time (Brond et al., 2021). Supporting this, retrospective genomic analysis of tumor samples from patients with stage I–II *versus* stage III–IV HGSOC revealed a higher frequency of whole-genome duplication in late-stage tumors compared to early-stage tumors (Cheng et al., 2022). Interestingly, copy number signatures appeared largely stable over time, from initial diagnosis through relapse or progression. These findings raise important questions about whether the TME differs in these patients. For example, patients with primary platinum resistance exhibited higher rates of CCNE1 and KRAS amplification at diagnosis, along with increased exposure to copy number signature 1 that is linked to a type of DNA instability known as breakage-fusion-bridge, which was negatively correlated with CD3 and CD8 expression (Smith et al., 2023).

This review highlights that targeting the replication stress response may induce a favorable shift in the TME. Serial tumor biopsies and paired peripheral blood mononuclear cell (PBMC) sampling can capture temporal tumor heterogeneity. To address this gap, patient-derived organoid cultures may serve as functional assays and facilitate the study of tumor–TME interactions. In preclinical models, fiber assays in organoids have been used to assess replication fork instability and predict sensitivity to prexasertib (a CHEK1 inhibitor) and VE-822 (an ATR inhibitor) (Hill et al., 2018); however, their reproducibility in clinical settings remains limited. Future studies should aim to develop tools capable of simultaneously evaluating replication stress and immune modulation in FFPE tissue or plasma.

The TME in HGSOC is particularly complex and unique; the peritoneal cavity provides a permissive niche for tumor dissemination through intricate interactions between metastatic tumor cells and TME components (Tan et al., 2006). Key cellular contributors include TAMs, cancer-associated adipocytes (CAAs), cancer-associated fibroblasts (CAFs), and cancer-associated mesothelial cells (CAMs), all of which play roles in promoting immune evasion (Tan et al., 2006). Additionally, a recently identified HGSOC subtype—C2 IGF2^+^ tumors—has been shown to engage fibroblasts via paracrine signaling, facilitating their transition into CAFs. This subtype is associated with stromal remodeling, genomic instability, stem-like features, and more advanced disease (Zhao et al., 2025).

A key area of investigation is how the TME may change in response to PARP or replication stress kinase inhibition, and the development of secondary resistance, and whether those changes promote immunosuppression through mechanisms such as senescence and activation of the STAT3 pathway, which increases expression of VEGF (Kamii et al., 2025; Zh et al., 2025; Martincuks et al., 2021b; Sumimoto et al., 2006). In addition to the immune microenvironment, angiogenesis is critical for tumor survival in hypoxic conditions, as high levels of VEGF promote the formation of abnormal vasculature that delivers oxygen and nutrients to cancer cells (Zhou et al., 2024). This pro-angiogenic TME has been associated with resistance to combinations of ATR inhibitors and anti–PD-L1 therapies (Kwon et al., 2022). Notably, triple therapy combining PARP inhibition, anti–PD-(L)1, and antiangiogenic agents has demonstrated clinical benefit in some clinical trials (Lee et al., 2025; Drew et al., 2024). However, whether this strategy can be extended to combinations involving ATR, CHK1, or WEE1 inhibitors remains unexplored.

The conventional on-and-off administration of replication stress kinase inhibitors may represent an interesting strategy to modulate the TME. Intermittent dosing enables active T cells to exert an anti-tumor response during the ‘off’ days, while selectively depleting exhausted T cells during the ‘on’ days; this approach could sensitize the cell to immunotherapies (Hardaker et al., 2024b).

Predictive biomarkers of response to replication stress kinase inhibitors and anti-PD-(L)1 therapies remain limited, in part due to the heterogeneity in HGSOC (Stur et al., 2025; Parvathareddy et al., 2021). However, studies have suggested that tumor immune infiltration and the expansion of CD8^+^ T cells may be associated with response to the combination of ceralasertib and durvalumab (Hardaker et al., 2024b). Interestingly, WEE1 inhibition has been shown to induce the recognition of endogenous retroviral RNA, leading to activation of interferon-stimulated genes (Brond et al., 2021). The presence of endogenous retrotransposable elements has been identified as a predictive biomarker of response to ICB in melanoma and non-small cell lung cancer (Herrera et al., 2025).

Our review aims to generate hypotheses and stimulate future research in HGSOC before immediate clinical application, given the initial disappointment of PD-1/PDL-1 in this disease. The dual-targeting approach focusing on replication stress response inhibition and anti–PD-(L)1 therapy—is based on mechanistic rationale and supported by emerging early-phase clinical trials in other tumor types. We acknowledge the limited availability of preclinical and clinical data specific to HGSOC and emphasize the need for the development of more representative preclinical models and clinical trial designs capable of capturing the dynamic changes in the tumor microenvironment, which could lead to the development of more effective treatment strategies.

## Glossary

ATM: Ataxia telangiectasia mutated
ATR: Ataxia telangiectasia and Rad3-related protein
ATRIP: ATR Interacting Protein
BID: Twice a day (bis in die)
BFB: Breakage–fusion–bridge
BRCA: Breast Cancer gene (1 or 2)
CAAs: Cancer-associated adipocytes
CAFs: Cancer-associated fibroblasts
CAMs: Cancer-associated mesothelial cells
CB: Clinical benefit
**CD8** ^**+**^ **T cells**: Cytotoxic T lymphocytes expressing CD8
CDK1/CDC2: Cyclin-dependent kinase 1
CDK2: Cyclin-dependent kinase 2
CCL5: C-C motif chemokine ligand 5
CCNE1: Cyclin E1
CIN: Chromosomal instability
cGAMP: Cyclic guanosine monophosphate–adenosine monophosphate
cGAS: Cyclic GMP-AMP synthase
CyTOF: Cytometry by Time Of Flight
DCs: Dendritic cells
DDR: DNA Damage Response
DNA: Deoxyribonucleic acid
ERVs: Endogenous retroviral elements
FFPE: Formalin-Fixed, Paraffin-Embedded
G1/S: Gap 1/Synthesis phase transition of the cell cycle
H3K9me3: Histone 3 lysine 9 trimethylation
HGSOC: High-grade serous ovarian cancer
HRD: Homologous recombination deficiency
HRR: Homologous recombination repair
IFN-I: Type I interferon
IL-6: Interleukin 6
IL-10: Interleukin 10
ID8: Mouse ovarian cancer cell line/model
ICB: Immune checkpoint blockade
IRF3: Interferon regulatory factor 3
LDG: Low-dose gemcitabine
MB49: Murine bladder carcinoma cell line/model
MHC I: Major histocompatibility complex class I
MHC II: Major histocompatibility complex class II
M-MDSCs: Monocytic myeloid-derived suppressor cells
MOS: Median overall survival
mPFS: Median progression-free survival
MYC: MYC proto-oncogene
NHEJ: Non-homologous end joining
NF1: Neurofibromin 1
NF-κB: Nuclear factor kappa B
ORR: Objective response rate
PARP: Poly (ADP-ribose) polymerase
PARPi: Poly (ADP-ribose) polymerase 1 and 2 inhibitors
PARG: Poly (ADP-ribose) glycohydrolase
PBMC: Peripheral blood mononuclear cell
PD-(L)1: Programmed death-(ligand) 1
PROC: Platinum-resistant ovarian cancer
RAS: Rat sarcoma (family of related GTPases; commonly mutated in cancer)
RB1: Retinoblastoma 1
RPA: Replication protein A
ROS: Reactive oxygen species
RSR: Replication stress response
S phase: Synthesis phase
scRNAseq: Single-cell RNA sequencing
siRNA: Small interfering RNA
SCLC: Small cell lung cancer
STAT3: Signal transducer and activator of transcription 3
STIC: Serous tubal intraepithelial carcinoma
STING: Stimulator of interferon genes
TBK1: TANK-binding kinase 1
TH1: T-helper 1 (immune response type)
TIM3: T-cell immunoglobulin and mucin-domain containing-3
TCR: T-cell receptor
TME: Tumor microenvironment
TP53: Tumor protein p53 (commonly abbreviated as p53)
Tregs: Regulatory T cells
TopBP1: Topoisomerase II Binding Protein 1
VEGF: Vascular endothelial growth factor
WEE1: WEE1 G2 checkpoint kinase
